# Learning Mindsets and Well-Being and Ill-Being Among Osteopathic Medical Students

**DOI:** 10.1001/jamanetworkopen.2024.18090

**Published:** 2024-06-14

**Authors:** Yoi Tibbetts, Zachary M. Himmelberger, Kenneth E. Barron, Mark R. Speicher, Chris S. Hulleman

**Affiliations:** 1 Motivate Lab, Harrisonburg, Virginia; 2 University of Virginia, Charlottesville; 3 James Madison University, Harrisonburg, Virginia; 4 American Association of Colleges of Osteopathic Medicine, Bethesda, Maryland

## Abstract

**Importance:**

Given the high rates of burnout and associated negative mental health outcomes (eg, depression, suicidal ideation, substance abuse) among medical students and physicians, it is imperative to identify strategies for supporting the future health workforce, particularly when considering trends indicating a future shortage of physicians. Understanding the associations of medical school students’ learning mindsets (eg, growth mindset, purpose and relevance, and sense of belonging) with indicators of well-being (eg, flourishing) and ill-being (eg, burnout) could provide a foundation for future research to consider when attempting to combat the negative mental health trends among medical students and physicians.

**Objectives:**

To understand the associations of medical school students’ learning mindsets (ie, their beliefs about themselves as learners and their learning environment) with critical student health outcomes (ie, well-being and ill-being).

**Design, Setting, and Participants:**

This survey study used a nationally representative sample of first-year osteopathic medical school students across the US who responded to a survey of learning mindsets as well as measures of well-being and ill-being in fall 2022. Data were analyzed from January to April 2024.

**Main Outcomes and Measures:**

Learning mindsets were categorized as growth mindset, purpose and relevance, and sense of belonging. Well-being was categorized as flourishing and resilience, and ill-being was categorized as burnout and psychological symptoms. Outcomes were regressed on learning mindset and demographics variables, and interactions of demographic variables and learning mindsets were assessed.

**Results:**

A total of 7839 students were surveyed, and 6622 students (mean [SD] age, 25.05 [3.20]; 3678 [55.5%] women) responded and were included in analyses. The 3 learning mindsets were significantly associated with flourishing (growth mindset: *b* = 0.34; 95% CI, 0.23 to 0.45; *P* < .001; purpose and relevance: *b* = 2.02; 95% CI, 1.83 to 2.20; *P* < .001; belonging uncertainty: *b* = −0.98; 95% CI, −1.08 to −0.89; *P* < .001) and resilience (growth mindset: *b* = 0.28; 95% CI, 0.17 to 0.40; *P* < .001; purpose and relevance: *b* = 1.62; 95% CI, 1.43 to 1.82; *P* < .001; belonging uncertainty: *b* = −1.50; 95% CI, −1.60 to −1.40; *P* < .001) well-being outcomes and burnout (growth mindset: *b* = −0.09; 95% CI, −0.11 to −0.07; *P* < .001; purpose and relevance: *b* = −0.29; 95% CI, −0.32 to −0.25; *P* < .001; belonging uncertainty: *b* = 0.28; 95% CI, 0.26 to 0.30; *P* < .001) and psychological symptoms (growth mindset: *b* = −0.22; 95% CI, −0.30 to −0.14; *P* < .001; purpose and relevance: *b* = −0.51; 95% CI, −0.64 to −0.38; *P* < .001; belonging uncertainty: *b* = 1.33; 95% CI, 1.27 to 1.40; *P* < .001) ill-being outcomes, even when controlling for important demographic characteristics (eg, race and ethnicity, gender identity, age). Furthermore, several significant interactions indicated that these learning mindsets may be particularly salient for students from historically marginalized communities: there was a significant interaction between growth mindset and race and ethnicity (*b* = 0.58; 95% CI, 0.08 to 1.09, *P* = .02), such that growth mindset was more strongly associated with flourishing among American Indian or Alaska Native, Black, Latine, or Native Hawaiian students.

**Conclusions and Relevance:**

These findings suggest that identifying strategies for supporting students’ learning mindsets may be an effective way to support medical student well-being and reduce ill-being, particularly among students from historically marginalized backgrounds.

## Key Points

**Question:**

Are medical school students’ learning mindsets associated with indicators of well-being and ill-being?

**Findings:**

This survey study including 6622 osteopathic medical school students found significant associations between learning mindsets and indicators of well-being and ill-being, with particularly strong associations among students from historically racially and ethnically marginalized populations.

**Meaning:**

These findings provide a foundation on which future research can investigate strategies designed to better support students’ learning mindsets and create more motivationally supportive learning environments that could benefit all students and students from historically marginalized backgrounds in particular.

## Teaser

This survey study examines the associations of medical school students’ learning mindsets (ie, their beliefs about themselves as learners and their learning environment) with well-being and ill-being, including measures of flourishing, resilience, burnout, and psychological symptoms.

## Introduction

Burnout is prevalent among US medical students, leading to a whole host of negative outcomes for the health workforce, including depression, suicidal ideation, and substance abuse.^1^ It is imperative to identify variables that can be systematically leveraged to create better environments for supporting our health workforce.^2^ Unfortunately, recent trends paint a glum picture. By 2034, it is estimated that there will be a shortage of 37 800 to 124 000 physicians in the US,^3^ due, in part, to burnout and suicide rates among physicians being double that of the general population.^4^ The purpose of this study was to identify potential ways of addressing burnout-related issues in future physicians by determining whether an association exists between student learning mindsets (ie, students’ beliefs about themselves as learners and their learning environment)^5^ and measures of well-being and ill-being in a national sample of osteopathic medical students. Establishing an association between learning mindsets in matriculating osteopathic medical students and well-being would suggest that interventions targeting the medical school environment could improve the well-being and success of future medical students and physicians.

This study focuses on 3 learning mindsets found to be associated with optimal learning and well-being: growth mindset, purpose and relevance, and sense of belonging.^5^ Growth mindset is the belief that intelligence and abilities are changeable rather than immutable. When students endorse a growth mindset, they recognize the important roles of effort, learning from mistakes, and pursuing mastery both in their time as trainees and as physicians.^6^ Medical students who perceive purpose and relevance in what they are learning often have increased motivation and higher ability to integrate and apply information, which has prompted schools to change to medical curriculum in ways that more readily highlight course relevance.^7^ When medical students experience a sense of belonging to a learning context, this can contribute to professional identity development,^8^ academic success,^9,10^ and choice of medical specialization in their future careers.^11^

We investigated these learning mindsets in particular because prior research has demonstrated that they are measurable, meaningful, particularly beneficial for students from historically underserved and marginalized backgrounds (eg, American Indian or Alaska Native, Black, Latine, or Native Hawaiian students and students who are the first in their family to complete a college education), and malleable.^12,13,14^ First, learning mindsets are measurable in that there are well-established, validated scales of each construct.^14^ Second, they are meaningful in that they are reliably predict critical academic performance and well-being outcomes.^15^ Third, educational practices that support learning mindsets are particularly beneficial for historically underserved students. For example, activities that help students find purpose and relevance in learning have been found to be especially beneficial for students from racially minoritized backgrounds and first-generation college students.^13^ Fourth, they are malleable in that shifts to be more supportive of learning mindsets have been reported to be associated with student outcomes,^16^ particularly for students from historically marginalized backgrounds.^12,13,17^ Learning mindset interventions also highlight the responsibility of the educational system (eg, administration) to create supportive environments, rather than placing the burden on students to adapt to an unsupportive environment.

As a result, growth mindset, purpose and relevance, and a sense of belonging provide opportunities to serve as levers that medical school faculty can use to better support students. However, since most research on learning mindsets has occurred in the kindergarten through 12th grade and undergraduate environments, it is important to establish the extent to which these learning mindsets are measurable, meaningful, and particularly important for the outcomes of students from historically marginalized backgrounds within the medical school context. If prior learning mindset findings can be replicated within the medical school context, it would indicate that future research should take advantage of the malleability of these constructs and target them as a promising strategy for supporting our future physician workforce.

## Methods

This survey study was deemed exempt from review by the WCG Clinical and Affiliates institutional review board. Participants provided informed consent at the onset of the entire entering student survey, which included additional items beyond just the student mindset and well-being sections. This study is reported following the American Association for Public Opinion Research (AAPOR) reporting guideline.

In 2021, the American Association of Colleges of Osteopathic Medicine (AACOM) received a grant from the Health Resources & Services Administration to develop an intervention targeted at improving the well-being of osteopathic medical school students. This federal funding permitted the selection and addition of a set of mindset and well-being questions to AACOM’s existing student survey. AACOM annually surveys matriculating students on their career plans, financial aid, medical school expectations, and demographic information (age, gender, race and ethnicity, and first-generation status). As part of this project, measures of learning mindsets, well-being, and ill-being were included in the fall 2022 survey for matriculating students. The full survey is published elsewhere.^18^

### Measures

Our analyses focused on 7 measures: 3 learning mindsets (growth mindset, purpose and relevance, and sense of belonging), 2 well-being measures (flourishing and resilience), and 2 ill-being measures (burnout and psychological symptoms associated with psychiatric disorders); the full scales for all measures are published elsewhere.^18^ Reliability statistics were calculated using the final sample. An expert panel of social psychologists and medical education researchers was used to identify salient measures of each of these constructs. Selected items were chosen as a result of a pragmatic measurement approach.^19^ That is, items were selected that demonstrated the ability to minimize the resources required for data collection (ie, fewer items would require less participant engagement) while maintaining the validity of the constructs being assessed. Prior research has suggested that the selected measures were valid in that they were associated with future outcomes, change as a result of experimental manipulations, and consistently maintained reliability over time.^12,13,14^

### Learning Mindsets

Growth mindset was measured using a 3-item (α = .92) scale adapted from Dweck et al.^20^ Purpose and relevance was measured using a 4-item scale (α = .87) adapted from Kosovich et al.^19^ Sense of belonging was measured using a 2-item (α = .83) scale adapted from the belonging uncertainty scale by Walton and Cohen.^21^ We chose to assess belonging uncertainty (the sense that one feels uncertain about whether or not they belong) rather than more positively framed belonging measures, as prior research has demonstrated that this feeling of doubt and uncertainty is more strongly associated with academic and well-being outcomes.^21,22^ Items from the 3 learning mindset scales were evaluated on a 6-point scale ranging from 1, indicating strongly disagree, to 6, strongly agree. Scores for each mindset were calculated (by reverse item-scoring when necessary) and we calculated the mean across the items, with higher scores indicating more of a growth mindset, greater purpose and relevance, or greater belonging uncertainty.

### Well-Being

Flourishing was measured using the 8-item (α = .92) Flourishing Scale.^23^ Items were evaluated on a 7-point scale ranging from 1, indicating strongly disagree, to 7, strongly agree. Resilience was assessed using a 10-item (α = .89), shortened version of the Connor-Davidson Resilience Scale.^24^ Items were evaluated on a 5-point scale ranging from 1, indicating not true at all, to 5, true nearly all the time. Scores for each well-being measure were calculated by reverse scoring when necessary and summing across items, so higher scores indicate greater flourishing or greater resilience.

### Ill-Being

Burnout was measured using a modified version of the 5-item (α = .88) emotional exhaustion subscale.^25^ Items were evaluated on a 5-point scale ranging from 1, indicating disagree strongly, to 5, agree strongly. Mean scores were calculated across items, such that higher scores indicated more burnout. Psychological symptoms were assessed using the 5-item (α = .81) Brief Symptom Rating Scale,^26^ which is designed for early identification of common psychiatric disorders (eg, depression and anxiety) and has been used as a screener for suicidal ideation in nonpsychiatric settings.^27^ Example items include rating your reaction to feeling down, and depressed, using a 5-point scale ranging from 1, indicating never, to 5, always or nearly always. Scores for each ill-being measure were calculated by summing across items, with higher scores indicating burnout or greater psychological symptoms.

### Demographic Measures

We assessed participants’ current gender identity using 8 potential categories (male, female, transgender male, transgender female, intersex, gender variant or nonconforming, not listed, and decline to answer). Participants were then grouped as (cisgender) male, (cisgender) female, or third gender (transgender male, transgender female, intersex, gender variant or nonconforming, or not listed). Race and ethnicity was assessed by allowing participants to select 1 or more identities. Participants were then grouped according to the Health Resources and Services Administration definition of underrepresented minority (ie, American Indian or Alaska Native, Black, Latine, or Native Hawaiian).^28^ If a participant had at least 1 underrepresented identity, they were included in that group. Participants were asked to identify the highest level of education attained by their parents. Students who had a parent with at least a 4-year undergraduate degree were considered continuing-generation students. All other participants were considered first-generation students.

### Statistical Analysis

For each outcome variable (flourishing, resilience, burnout, and psychological symptoms), we created 2 models. In the first model, the outcome was regressed on growth mindset, purpose and relevance, belonging uncertainty, age, gender (male vs female), race and ethnicity (American Indian or Alaska Native, Black, Latine, or Native Hawaiian vs Asian or White), and first-generation status (first generation vs continuing generation). All categorical variables were dummy-coded and all quantitative variables were mean-centered. The second model contained the aforementioned terms and all 2-way interactions involving demographic variables, 2-way interactions involving 1 demographic variable and a learning mindset, and 3-way interactions involving 2 demographic variables and a learning mindset. We included this set of interactions based on previous research^13,14^ and to explore how intersections of student identities are associated with well-being and ill-being. Not including the opportunity for demographic factors to interact could lead to an oversimplification of an individual’s identity that can produce a limited understanding of their actual experience.^29^ By allowing various demographic factors to interact, we are able to investigate the student experience with more nuance.^13,30^ We evaluated interactions in 2 ways. First, we assessed the overall impact of adding interactions by using an analysis of variance to compare the fit of models with and without interaction terms. Second, regardless of improvements to model fit, we examined the model coefficients to explore how preexisting demographic outcomes were moderated by learning mindsets. All statistical tests were 2-sided and evaluated using a significance level *P* < .05. Analyses were conducted using R software version 4.2.1 (R Project for Statistical Computing). Data were analyzed from January to April 2024.

## Results

### Respondents and Data Screening

Of 9169 entering students who received the survey request, 7839 (85%) responded to at least 1 part of the survey. Of these respondents, 6666 students provided demographic information and responded to the additional learning mindsets, well-being, and ill-being items. To explore the intersectionality between different identities, we excluded 44 respondents who reported demographic categories that would have yielded small sample sizes for statistical power (eg, few participants identified as a third gender and American Indian or Alaska Native, Black, Latine, or Native Hawaiian). Our final sample included 6622 students (mean [SD] age, 25.05 [3.20]; 3678 [55.5%] women), reflecting 72% of all matriculating osteopathic medical students in the class of 2026. Overall, 0.1% of students were American Indian, 25.1% of students were Asian, 3.8% of students were Black, 7.9% of students were Latine of any race, less than 0.1% of students were Pacific Islander, 57.4% of students were White, and 5.7% of students reported 2 or more races or ethnicities; 31.8% of American Indian or Alaska Native, Black, Latine, or Native Hawaiian students and 18.2% of Asian or White students were first-generation students. Age was studied in the event there were systematic differences in well-being and ill-being measures between younger students (who, for example, may have very brief or no work experiences) and older students (who are more likely to have work experiences). Descriptive and correlational statistics are presented in Table 1. We found no significant differences when examining how the demographics included in the study sample differed from the entire pool of matriculating medical students in fall 2022, indicating that our sample was nationally representative.

**Table 1.** Correlation Matrix and Descriptive Statistics by Demographics

Measure	Growth mindset	Purpose and relevance	Belonging uncertainty	Flourishing	Resilience	Burnout	Psychological symptoms
Measure correlation, *r*							
Growth mindset	1	NA	NA	NA	NA	NA	NA
Purpose & relevance	0.19	1	NA	NA	NA	NA	NA
Belonging uncertainty	−0.18	−0.20	1	NA	NA	NA	NA
Flourishing	0.16	0.31	−0.29	1	NA	NA	NA
Resilience	0.15	0.26	−0.41	0.44	1	NA	NA
Burnout	−0.20	−0.28	0.42	−0.35	−0.33	1	NA
Psychological symptoms	−0.15	−0.18	0.49	−0.35	−0.37	0.59	1
Scores by demographic variables, mean (SE)							
Total (N = 6666)	4.71 (0.01)	5.44 (0.01)	3.31 (0.01)	46.55 (0.06)	31.39 (0.07)	2.24 (0.01)	7.46 (0.04)
Gender							
Female (n = 3678)	4.76 (0.02)	5.45 (0.01)	3.52 (0.02)	46.70 (0.08)	30.93 (0.09)	2.26 (0.02)	7.93 (0.06)
Male (n = 2944)	4.64 (0.02)	5.42 (0.01)	3.05 (0.02)	46.40 (0.10)	31.99 (0.10)	2.20 (0.02)	6.85 (0.07)
Third gender (n = 44)	4.85 (0.14)	5.33 (0.10)	3.65 (0.18)	43.77 (1.11)	29.50 (0.99)	2.48 (0.13)	8.64 (0.66)
Race and ethnicity^a^							
Asian (n = 1670)	4.71 (0.02)	5.37 (0.02)	3.40 (0.03)	45.22 (0.13)	29.98 (0.14)	2.37 (0.02)	7.60 (0.09)
Black (n = 255)	4.95 (0.06)	5.50 (0.04)	3.15 (0.08)	47.13 (0.32)	31.64 (0.32)	2.15 (0.06)	6.11 (0.22)
Latine (of any race) (n = 528)	4.84 (0.04)	5.48 (0.03)	3.25 (0.06)	46.78 (0.21)	31.71 (0.23)	2.19 (0.04)	7.41 (0.17)
≥2 races or ethnicities (n = 377)	4.73 (0.05)	5.41 (0.03)	3.41 (0.06)	46.63 (0.26)	31.35 (0.28)	2.30 (0.05)	7.84 (0.19)
White (n = 3828)	4.67 (0.02)	5.46 (0.01)	3.28 (0.02)	47.04 (0.08)	31.94 (0.08)	2.19 (0.01)	7.46 (0.06)
Family education history							
Continuing generation (n = 5319)	4.68 (0.01)	5.42 (0.01)	3.32 (0.02)	46.54 (0.07)	31.28 (0.07)	2.24 (0.01)	7.51 (0.05)
First generation (n = 1347)	4.82 (0.03)	5.48 (0.02)	3.28 (0.03)	46.57 (0.13)	31.80 (0.15)	2.21 (0.03)	7.23 (0.10)

Abbreviation: NA, not applicable.

^a^ Students with a racial or ethnic background with 5 or fewer respondents were censored to protect anonymity.

### Well-Being Outcomes

**Figure 1.**
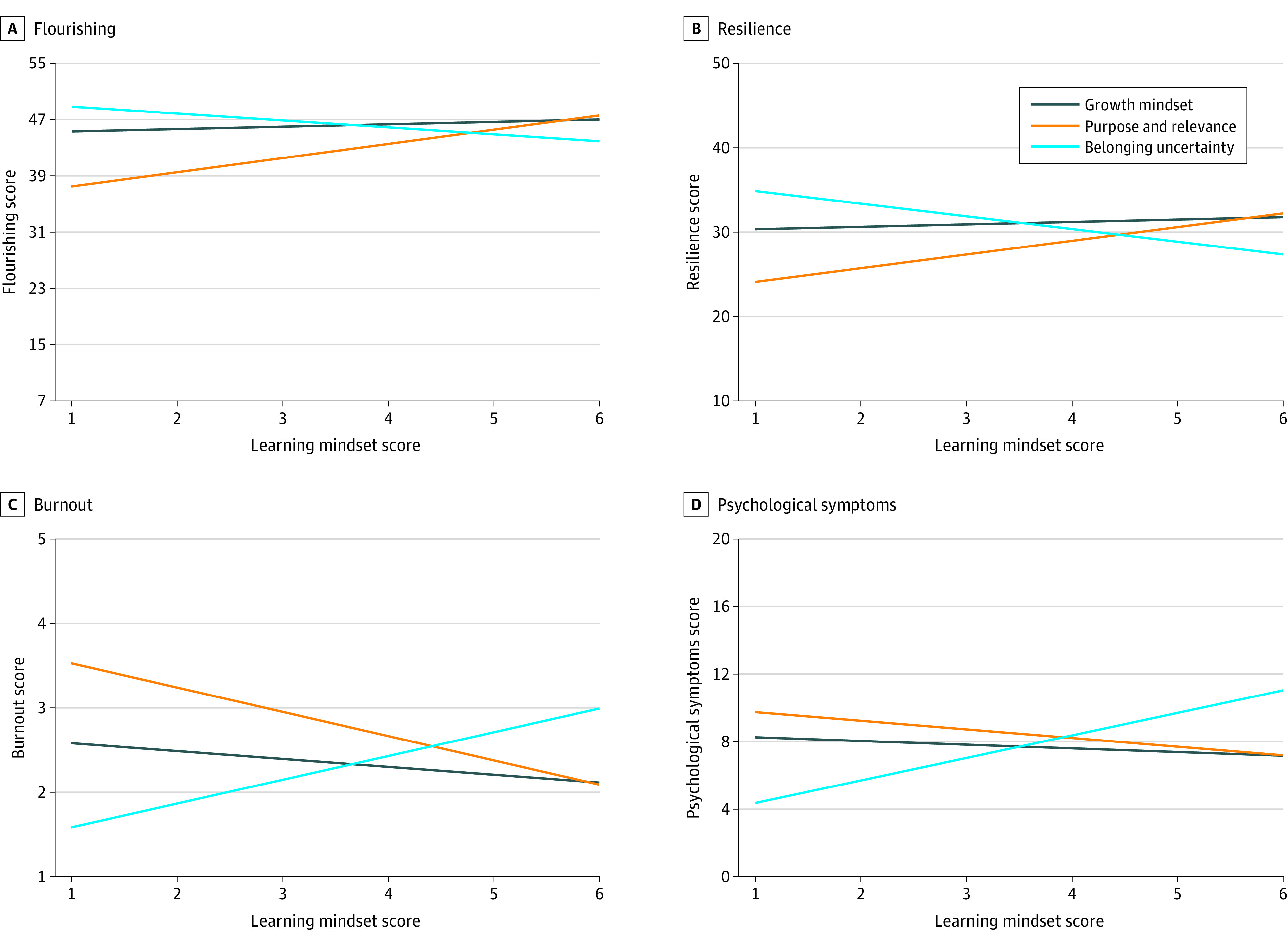
Marginal Effects for the Associations of Learning Mindsets With Outcomes Marginal effects were calculated after accounting for the other variables in the models.

**Figure 2.**
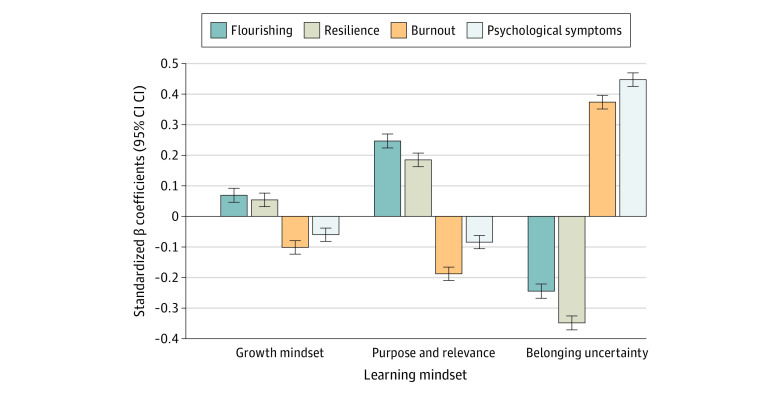
Standardized Regression Coefficients for the Associations of Learning Mindsets With Outcomes

Each of the learning mindsets was significantly associated with both well-being outcomes (Table 2, Figure 1, and Figure 2). Specifically, growth mindset and purpose and relevance were positively associated with flourishing (growth mindset: *b* = 0.34; 95% CI, 0.23 to 0.45; *P* < .001; purpose and relevance: *b* = 2.02; 95% CI, 1.83 to 2.20; *P* < .001) and resilience (growth mindset: *b* = 0.28; 95% CI, 0.17 to 0.40); *P* < .001; purpose and relevance: *b* = 1.62; 95% CI, 1.43 to 1.82; *P* < .001), whereas higher levels of belonging uncertainty was a risk factor associated with lower flourishing (*b* = −0.98; 95% CI, −1.08 to −0.89; *P* < .001) and resilience (*b* = −1.50; 95% CI, −1.60 to −1.40; *P* < .001). Furthermore, female students reported significantly higher levels of flourishing than male students (*b* = −0.66; 95% CI, −0.88 to −0.43; *P* < .001), whereas older students (*b* = 0.07; 95% CI, 0.04 to 0.11; *P* < .001) and male students (*b* = 0.38; 95% CI, 0.15 to 0.62; *P* = .001) reported significantly more resilience. Adding the interaction terms resulted in a significantly improved model fit for resilience (change in *F*_36, 6578_ = 1.59; *P* = .01) but not for flourishing (change in *F*_36, 6578_ = 1.19; *P* = .20).

**Table 2.** Regression Model Coefficients

Term	Flourishing^a^			Resilience^b^			Burnout^c^			Psychological symptoms^d^			
	β	*b* (95% CI)	*P* value	β	*b* (95% CI)	*P* value	β	*b* (95% CI)	*P* value	β	b (95% CI)	*P* value
Intercept	0.00	46.87 (46.71 to 47.03)	<.001	0.00	31.18 (31.01 to 31.35)	<.001	0.00	2.21 (2.18 to 2.24)	<.001	0.00	7.77 (7.66 to 7.89)	<.001
Age	0.00	0.00 (−0.03 to 0.04)	.99	0.04	0.07 (0.04 to 0.11)	<.001	−0.00	−0.00 (−0.01 to 0.00)	.66	0.04	0.04 (0.02 to 0.07)	<.001
Male	-0.07	−0.66 (−0.88 to −0.43)	<.001	0.04	0.38 (0.15 to 0.62)	.001	0.03	0.05 (0.01 to 0.09)	.01	−0.07	−0.53 (−0.69 to −0.37)	<.001
American Indian or Alaska Native, Black, Latine, or Native Hawaiian race or ethnicity	0.01	0.09 (−0.22 to 0.41)	.56	−0.00	−0.00 (−0.33 to 0.32)	.98	−0.00	−0.01 (−0.07 to 0.05)	.72	−0.03	−0.34 (−0.55 to −0.12)	.003
First generation college graduate	-0.01	−0.14 (−0.41 to 0.14)	.33	0.02	0.26 (−0.03 to 0.54)	.08	0.01	0.01 (−0.04 to 0.06)	.64	−0.02	−0.18 (−0.37 to 0.02)	.07
Growth mindset	0.07	0.34 (0.23 to 0.45)	<.001	0.05	0.28 (0.17 to 0.40)	<.001	−0.10	−0.09 (−0.11 to −0.07)	<.001	−0.06	−0.22 (−0.30 to −0.14)	<.001
Purpose and relevance	0.25	2.02 (1.83 to 2.20)	<.001	0.18	1.62 (1.43 to 1.82)	<.001	−0.19	−0.29 (−0.32 to −0.25)	<.001	−0.08	−0.51 (−0.64 to −0.38)	<.001
Belonging uncertainty	−0.24	−0.98 (−1.08 to −0.89)	<.001	−0.35	−1.50 (−1.60 to −1.40)	<.001	0.37	0.28 (0.26 to 0.30)	<.001	0.45	1.33 (1.27 to 1.40)	<.001

^a^ *R*^2^ = 0.161; root mean square error, 4.52.

^b^ *R*^2^ = 0.207; root mean square error, 4.72.

^c^ *R*^2^ = 0.231; root mean square error, 0.81.

^d^ *R*^2^ = 0.253; root mean square error, 3.17.

### Ill-Being Outcomes

Learning mindsets were also significantly associated with both ill-being outcomes (Table 2, Figure 1, and Figure 2). Specifically, growth mindset and purpose and relevance were negatively associated with burnout (growth mindset: *b* = −0.09; 95% CI, −0.11 to −0.07; *P* < .001; purpose and relevance: *b* = −0.29; 95% CI, −0.32 to −0.25; *P* < .001) and psychological symptoms (growth mindset: *b* = −0.22; 95% CI, −0.30 to −0.14; *P* < .001; purpose and relevance: *b* = −0.51; 95% CI, −0.64 to −0.38; *P* < .001) and higher levels of belonging uncertainty was a risk factor associated with higher burnout (*b* = 0.28; 95% CI, 0.26 to 0.30; *P* < .001) and more psychological symptoms (*b* = 1.33; 95% CI, 1.27 to 1.40; *P* < .001). Furthermore, although female students reported higher burnout scores at an overall mean level, once we controlled for other demographic factors and learning mindsets in regression analyses, male students had higher burnout scores. Female students, older students, and Asian and White students reported significantly higher levels of psychological symptoms (Table 2). Adding the interaction terms resulted in a significantly improved model fit for psychological symptoms (change in*F*_36, 6578_ = 1.44; *P* = .04) but not for burnout (change in *F*_36, 6578_ = 1.23; *P* = .16).

### Significant Interactions

Several significant interactions indicated that learning mindsets were associated with particularly beneficial outcomes for the well-being and ill-being of students from historically marginalized backgrounds. For example, the flourishing of students from American Indian or Alaska Native, Black, Latine, or Native Hawaiian backgrounds was more strongly related to having a growth mindset (growth mindset × race and ethnicity interaction: *b* = 0.58; 95% CI, 0.08 to 1.09, *P* = .02). Similarly, the more purpose and relevance students from marginalized groups perceived, the less likely they were to report psychological symptoms (purpose and relevance × race and ethnicity interaction: *b* = –0.91; 95% CI, –1.49 to –0.34, *P* = .002). Thus, a critical finding from this study is that although growth mindset, purpose and relevance, and belonging uncertainty were significantly associated with all of the outcomes in this study for all students, they were even more strongly associated with outcomes for students from historically marginalized backgrounds. Full models, including all interactions, are provided in eTables 1 to 4 and eFigures 1 to 14 in Supplement 1.

## Discussion

This survey study found that within osteopathic medical schools, these learning mindsets are measurable (ie, we successfully captured student reports of growth mindset, purpose and relevance, and sense of belonging), meaningful (ie, our measures of learning mindsets were all significantly associated with validated measures of well-being and ill-being), and particularly beneficial for students from historically marginalized backgrounds (ie, we found significant interactions indicating that adaptive learning mindsets were more strongly associated with well-being and ill-being outcomes among American Indian or Alaska Native, Black, Latine, or Native Hawaiian students). The next logical step is to investigate the malleability of these constructs within the medical school context.

The findings of this study lay a foundation for future work to build on to take advantage of the power of learning mindsets and maximize their potential for supporting future medical students and physicians. Indeed, some learning mindsets were more strongly associated with outcomes than others. For example, our data indicate that a 1-unit increase in belonging uncertainty was associated with an additional 1.33 psychological symptoms, making it a particularly notable risk factor. With national trends indicating that the number of medical educators in the US is declining, along with their overall funding,^31^ it is imperative that the field identifies strategies, based on reliable data, to better support this critical workforce.

### Limitations

This study has some limitations. One limitation of our study is that we were working with correlational, cross-sectional data. Because the learning mindset data were not collected prior to assessing well-being and ill-being, significant associations cannot be interpreted as causal. Evidence for causal relationships between learning mindsets and well-being and ill-being would need to be established via more robust methods (eg, experimental protocols that manipulated learning mindsets as independent variables and then measured changes in well-being and ill-being as dependent variables).

Additionally, although we started investigating how students’ intersectional identities (eg, considering the intersections of race and generational status) are associated with learning mindsets and well-being and ill-being, the inclusion of race and ethnicity in our analytic models was crude in that it only divided students into 2 groups: either American Indian or Alaska Native, Black, Latine, or Native Hawaiian or White or Asian. The inclusion of race and ethnicity and social class indicators (eg, generational status) in our models represents an initial step toward exploring students’ intersectional identities, but a more robust set of analyses would further disaggregate race and ethnicity. For power considerations, we were unable to conduct these analyses within the present sample, but our plan is to continue to collect data so that these important analyses are feasible in future research.

Furthermore, the present findings are based exclusively on first-year medical students. Greater insight could be gleaned from a repeated-measures design that tracked these constructs over time. Conclusions based on a 1-time survey of first-year medical students may be underestimating the extent to which medical students experience burnout throughout medical school.

## Conclusions

In this survey study, we found a significant association between measurable learning mindsets and medical student well-being and ill-being. Furthermore, extensive research has demonstrated that when contexts support these learning mindsets, it often results in improved academic and mental health outcomes.^16^ Indeed, these constructs are malleable and amenable to intervention.^15^ We contend that identifying strategies that leverage learning mindsets in the context of medical school has the potential to support a healthier and more diverse physician pool.

## Notes

Supplement 1. **eTable 1.** Flourishing Interaction Model Coefficients **eTable 2.** Resilience Interaction Model Coefficients **eTable 3.** Burnout Interaction Model Coefficients **eTable 4.** Psychological Symptoms Interaction Model Coefficients **eFigure 1.** Ethnicity × Growth Mindset for Flourishing **eFigure 2.** Gender × Purpose for Flourishing **eFigure 3.** Ethnicity × Gender for Resilience **eFigure 4.** Gender × Growth Mindset for Resilience **eFigure 5.** Gender × First Generation Status × Purpose for Resilience **eFigure 6.** Age × Belonging Uncertainty for Burnout **eFigure 7.** Age × Gender × Purpose for Burnout **eFigure 8.** Gender × First Generation Status × Purpose for Burnout **eFigure 9.** Age × Gender for Psychological Symptoms **eFigure 10.** Age × Ethnicity for Psychological Symptoms **eFigure 11.** Age × Growth Mindset for Psychological Symptoms **eFigure 12.** Ethnicity × Purpose for Psychological Symptoms **eFigure 13.** Age × Gender × Purpose for Psychological Symptoms **eFigure 14.** Ethnicity × First Generation Status × Purpose for Psychological Symptoms

Supplement 2.
Data Sharing Statement
