# Duplicate Paragraph in Results

**DOI:** 10.1001/jamanetworkopen.2024.27297

**Published:** 2024-07-12

In the Original Investigation titled “Learning Mindsets and Well-Being and Ill-Being Among Osteopathic Medical Students,”^1^ published June 14, 2024, there was a duplicate paragraph at the end of the Results section. This article has been corrected.^1^

## References

[1] Tibbetts Y, Himmelberger ZM, Barron KE, Speicher MR, Hulleman CS. Learning mindsets and well-being and ill-being among osteopathic medical students. JAMA Netw Open. 2024;7(6):e2418090. doi:10.1001/jamanetworkopen.2024.18090 38874920 PMC11179131

