# Supplementary material for: Learning Mindsets and Well-Being and Ill-Being Among Osteopathic Medical Students
Source: JAMA Netw Open. 2024 Jun 14;7(6):e2418090. doi: 10.1001/jamanetworkopen.2024.18090 (PMC11179131; doi:10.1001/jamanetworkopen.2024.18090)
Supplement: Supplement 1. — eTable 1. Flourishing Interaction Model Coefficients eTable 2. Resilience Interaction Model Coefficients eTable 3. Burnout Interaction Model Coefficients eTable 4. Psychological Symptoms Interaction Model Coefficients eFigure 1. Ethnicity × Growth Mindset for Flourishing eFigure 2. Gender × Purpose for Flourishing eFigure 3. Ethnicity × Gender for Resilience eFigure 4. Gender × Growth Mindset for Resilience eFigure 5. Gender × First Generation Status × Purpose for Resilience eFigure 6. Age × Belonging Uncertainty for Burnout eFigure 7. Age × Gender × Purpose for Burnout eFigure 8. Gender × First Generation Status × Purpose for Burnout eFigure 9. Age × Gender for Psychological Symptoms eFigure 10. Age × Ethnicity for Psychological Symptoms eFigure 11. Age × Growth Mindset for Psychological Symptoms eFigure 12. Ethnicity × Purpose for Psychological Symptoms eFigure 13. Age × Gender × Purpose for Psychological Symptoms eFigure 14. Ethnicity × First Generation Status × Purpose for Psychological Symptoms [file jamanetwopen-e2418090-s001.pdf]

Supplemental Online Content

Tibbetts Y, Himmelberger ZM, Barron KE, Speicher MR, Hulleman CS. Learning mindsets and well-being and ill-being among osteopathic medical students. JAMA Netw Open. 2024;7(6):e2418090. doi:10.1001/jamanetworkopen.2024.18090

eTable 1. Flourishing Interaction Model Coefficients
eTable 2. Resilience Interaction Model Coefficients
eTable 3. Burnout Interaction Model Coefficients
eTable 4. Psychological Symptoms Interaction Model Coefficients
eFigure 1. Ethnicity × Growth Mindset for Flourishing
eFigure 2. Gender × Purpose for Flourishing
eFigure 3. Ethnicity × Gender for Resilience
eFigure 4. Gender × Growth Mindset for Resilience
eFigure 5. Gender × First Generation Status × Purpose for Resilience
eFigure 6. Age × Belonging Uncertainty for Burnout
eFigure 7. Age × Gender × Purpose for Burnout
eFigure 8. Gender × First Generation Status × Purpose for Burnout
eFigure 9. Age × Gender for Psychological Symptoms
eFigure 10. Age × Ethnicity for Psychological Symptoms
eFigure 11. Age × Growth Mindset for Psychological Symptoms
eFigure 12. Ethnicity × Purpose for Psychological Symptoms
eFigure 13. Age × Gender × Purpose for Psychological Symptoms
eFigure 14. Ethnicity × First Generation Status × Purpose for Psychological Symptoms

This supplemental material has been provided by the authors to give readers additional information about their work.

© 2024 Tibbetts Y et al. *JAMA Network Open.*

eTable 1. Flourishing Interaction Model Coefficients

Terms	*β*	*b*	*95% CI*	*p*
Intercept	0.00	46.84	46.66, 47.02	< .001
Age	-0.01	-0.01	-0.07, 0.04	.65
Male	-0.06	-0.63	-0.90, -0.37	< .001
URiM	-0.01	-0.15	-0.61, 0.32	.53
First Gen.	0.00	0.03	-0.39, 0.44	.90
Growth Mindset	0.06	0.29	0.11, 0.48	.002
Purpose	0.20	1.67	1.36, 1.98	< .001
Belong. Uncertain.	-0.25	-0.99	-1.14, -0.83	< .001
Age X Male	0.00	0.00	-0.07, 0.08	.91
Age X URiM	0.00	-0.01	-0.11, 0.09	.89
Age X First Gen.	0.01	0.03	-0.05, 0.12	.46
Male X URiM	0.01	0.30	-0.37, 0.97	.38
Male X First Gen.	-0.02	-0.31	-0.88, 0.27	.30
URiM X First Gen.	0.00	0.06	-0.66, 0.78	.88
Age X Growth Mindset	-0.01	-0.01	-0.06, 0.04	.71
Male X Growth Mindset	-0.01	-0.05	-0.31, 0.22	.73
URiM X Growth Mindset	0.05	0.58	0.08, 1.09	.02
First Gen. X Growth Mindset	0.00	0.02	-0.44, 0.47	.94
Age X Purpose	-0.01	-0.03	-0.13, 0.06	.50
Male X Purpose	0.06	0.67	0.24, 1.11	.003
URiM X Purpose	0.02	0.50	-0.33, 1.32	.24
First Gen. X Purpose	0.01	0.28	-0.47, 1.04	.47
Age X Belong. Uncertain.	-0.03	-0.04	-0.09, 0.01	.12
Male X Belong. Uncertain.	-0.01	-0.06	-0.29, 0.16	.59
URiM X Belong. Uncertain.	0.02	0.16	-0.23, 0.56	.41
First Gen. X Belong. Uncertain.	0.01	0.09	-0.26, 0.44	.61
Age X Male X Growth Mindset	0.02	0.04	-0.02, 0.11	.19
Age X URiM X Growth Mindset	0.00	0.01	-0.08, 0.10	.77
Age X First Gen. X Growth Mindset	-0.03	-0.07	-0.15, 0.00	.06
Male X URiM X Growth Mindset	-0.02	-0.42	-1.10, 0.25	.22
Male X First Gen. X Growth Mindset	0.02	0.32	-0.28, 0.91	.29
URiM X First Gen. X Growth Mindset	-0.01	-0.28	-1.03, 0.47	.47
Age X Male X Purpose	-0.01	-0.04	-0.16, 0.09	.57
Age X URiM X Purpose	-0.03	-0.12	-0.27, 0.03	.13
Age X First Generation X Purpose	0.03	0.12	-0.03, 0.27	.13
Male X URiM X Purpose	-0.01	-0.43	-1.55, 0.69	.45
Male X First Gen. X Purpose	-0.00	-0.1	-1.12, 0.92	.85
URiM X First Gen. X Purpose	-0.03	-1.05	-2.35, 0.25	.12
Age X Male X Belong. Uncertainty	0.01	0.01	-0.05, 0.07	.81
Age X URiM X Belong. Uncertain.	0.00	-0.01	-0.08, 0.07	.83
Age X First Gen. X Belong. Uncertain.	0.02	0.05	-0.02, 0.11	.16
Male X URiM X Belong. Uncertain.	-0.02	-0.31	-0.85, 0.24	.27
Male X First Gen. X Belong. Uncertain.	0.01	0.18	-0.30, 0.66	.47
URiM X First Gen. X Belong. Uncertain.	-0.02	-0.27	-0.85, 0.32	.37

Note: R^2 = .167; *RMSE* = 4.51; URiM = underrepsented in medicine (i.e., Black/Latine/Indigenous students; Belong. Uncertain. = belonging uncertainty; *b* = unstandardized beta coefficient; β = standardized beta coefficient; *RMSE* = root mean square error

© 2024 Tibbetts Y et al. *JAMA Network Open.*

eTable 2. Resilience Interaction Model Coefficients

Terms	*β*	*b*	*95% CI*	*p*
Intercept	0.00	31.21	31.02, 31.40	< .001
Age	0.07	0.11	0.05, 0.18	< .001
Male	0.03	0.28	0.00, 0.56	.05
URiM	-0.03	-0.46	-0.95, 0.02	.06
First Gen.	0.01	0.18	-0.25, 0.61	.41
Growth Mindset	0.07	0.36	0.17, 0.55	< .001
Purpose	0.19	1.63	1.31, 1.95	< .001
Belong. Uncertain.	-0.34	-1.49	-1.65, -1.32	< .001
Age X Male	-0.03	-0.08	-0.16, 0.00	.05
Age X URiM	-0.01	-0.04	-0.15, 0.06	.40
Age X First Gen.	0.01	0.02	-0.07, 0.11	.68
Male X URiM	0.03	0.74	0.04, 1.44	.04
Male X First Gen.	0.00	0.01	-0.59, 0.61	.98
URiM X First Gen.	0.01	0.29	-0.46, 1.04	.45
Age X Growth Mindset	-0.02	-0.02	-0.07, 0.03	.41
Male X Growth Mindset	-0.06	-0.42	-0.70, -0.15	.003
URiM X Growth Mindset	0.04	0.49	-0.04, 1.02	.07
First Gen. X Growth Mindset	0.02	0.28	-0.20, 0.75	.25
Age X Purpose	-0.03	-0.07	-0.17, 0.03	.18
Male X Purpose	-0.01	-0.18	-0.63, 0.28	.44
URiM X Purpose	0.03	0.59	-0.27, 1.45	.18
First Gen. X Purpose	0.03	0.64	-0.15, 1.43	.11
Age X Belong. Uncertain.	-0.02	-0.03	-0.08, 0.03	.32
Male X Belong. Uncertain.	-0.02	-0.10	-0.33, 0.13	.39
URiM X Belong. Uncertain.	0.04	0.39	-0.02, 0.81	.06
First Gen. X Belong. Uncertain.	0.00	0.03	-0.34, 0.40	.87
Age X Male X Growth Mindset	0.02	0.04	-0.03, 0.10	.28
Age X URiM X Growth Mindset	-0.01	-0.03	-0.12, 0.06	.50
Age X First Gen. X Growth Mindset	-0.01	-0.01	-0.09, 0.07	.73
Male X URiM X Growth Mindset	0.01	0.21	-0.50, 0.91	.57
Male X First Gen. X Growth Mindset	0.01	0.12	-0.50, 0.74	.70
URiM X First Gen. X Growth Mindset	-0.01	-0.21	-0.99, 0.58	.60
Age X Male X Purpose	0.03	0.10	-0.04, 0.23	.16
Age X URiM X Purpose	-0.03	-0.09	-0.25, 0.06	.25
Age X First Generation X Purpose	0.01	0.05	-0.11, 0.21	.54
Male X URiM X Purpose	-0.02	-0.82	-1.98, 0.35	.17
Male X First Gen. X Purpose	0.00	0.08	-0.98, 1.14	.89
URiM X First Gen. X Purpose	-0.04	-1.72	-3.07, -0.36	.01
Age X Male X Belong. Uncertainty	0.01	0.02	-0.04, 0.09	.49
Age X URiM X Belong. Uncertain.	-0.01	-0.02	-0.10, 0.06	.59
Age X First Gen. X Belong. Uncertain.	0.00	0.01	-0.06, 0.08	.81
Male X URiM X Belong. Uncertain.	-0.02	-0.39	-0.96, 0.17	.17
Male X First Gen. X Belong. Uncertain.	0.00	0.02	-0.48, 0.52	.94
URiM X First Gen. X Belong. Uncertain.	-0.00	-0.06	-0.67, 0.55	.85

Note: R^2 = .214; *RMSE* = 4.70; URiM = underrepsented in medicine (i.e., Black/Latine/Indigenous students; Belong. Uncertain. = belonging uncertainty; *b* = unstandardized beta coefficient; β = standardized beta coefficient; *RMSE* = root mean square error

© 2024 Tibbetts Y et al. *JAMA Network Open.*

eTable 3. Burnout Interaction Model Coefficients

Terms	*β*	*b*	*95% CI*	*p*
Intercept	0.00	2.22	2.18, 2.25	< .001
Age	0.01	0.00	-0.01, 0.01	.64
Male	0.03	0.05	0.00, 0.10	.05
URiM	-0.01	-0.04	-0.12, 0.05	.40
First Gen.	0.01	0.01	-0.06, 0.09	.73
Growth Mindset	-0.12	-0.11	-0.15, -0.08	< .001
Purpose	-0.19	-0.29	-0.35, -0.24	< .001
Belong. Uncertain.	0.37	0.28	0.25, 0.31	< .001
Age X Male	-0.01	0.00	-0.02, 0.01	.70
Age X URiM	-0.03	-0.02	-0.04, 0.00	.05
Age X First Gen.	0.01	0.01	-0.01, 0.02	.46
Male X URiM	0.01	0.05	-0.07, 0.17	.43
Male X First Gen.	-0.01	-0.02	-0.12, 0.08	.69
URiM X First Gen.	0.01	0.04	-0.08, 0.17	.50
Age X Growth Mindset	0.00	0.00	-0.01, 0.01	.95
Male X Growth Mindset	0.02	0.03	-0.02, 0.08	.20
URiM X Growth Mindset	0.01	0.01	-0.08, 0.11	.76
First Gen. X Growth Mindset	0.02	0.05	-0.03, 0.13	.26
Age X Purpose	0.03	0.01	-0.01, 0.03	.17
Male X Purpose	0.00	0.01	-0.07, 0.08	.87
URiM X Purpose	-0.01	-0.05	-0.20, 0.10	.49
First Gen. X Purpose	-0.03	-0.12	-0.26, 0.01	.08
Age X Belong. Uncertain.	-0.04	-0.01	-0.02, 0.00	.04
Male X Belong. Uncertain.	0.00	0.00	-0.04, 0.04	.89
URiM X Belong. Uncertain.	-0.01	-0.02	-0.09, 0.05	.58
First Gen. X Belong. Uncertain.	-0.01	-0.01	-0.08, 0.05	.68
Age X Male X Growth Mindset	0.02	0.01	0.00, 0.02	.18
Age X URiM X Growth Mindset	-0.01	-0.01	-0.02, 0.01	.50
Age X First Gen. X Growth Mindset	0.00	0.00	-0.01, 0.02	.76
Male X URiM X Growth Mindset	0.00	0.01	-0.11, 0.13	.83
Male X First Gen. X Growth Mindset	-0.03	-0.09	-0.20, 0.01	.09
URiM X First Gen. X Growth Mindset	0.00	0.00	-0.14, 0.13	.96
Age X Male X Purpose	-0.06	-0.03	-0.05, -0.01	.007
Age X URiM X Purpose	0.02	0.02	-0.01, 0.04	.25
Age X First Generation X Purpose	-0.01	-0.01	-0.03, 0.02	.68
Male X URiM X Purpose	0.02	0.11	-0.09, 0.31	.29
Male X First Gen. X Purpose	0.04	0.22	0.04, 0.40	.02
URiM X First Gen. X Purpose	0.03	0.21	-0.02, 0.44	.08
Age X Male X Belong. Uncertainty	0.02	0.01	-0.01, 0.02	.31
Age X URiM X Belong. Uncertain.	-0.01	0.00	-0.02, 0.01	.56
Age X First Gen. X Belong. Uncertain.	0.03	0.01	0.00, 0.02	.10
Male X URiM X Belong. Uncertain.	0.01	0.01	-0.08, 0.11	.77
Male X First Gen. X Belong. Uncertain.	0.00	0.01	-0.08, 0.09	.90
URiM X First Gen. X Belong. Uncertain.	0.02	0.07	-0.04, 0.17	.20

Note: R^2 = .259; *RMSE* = 3.15; URiM = underrepsented in medicine (i.e., Black/Latine/Indigenous students; Belong. Uncertain. = belonging uncertainty; *b* = unstandardized beta coefficient; *β* = standardized beta coefficient; *RMSE* = root mean square error

© 2024 Tibbetts Y et al. *JAMA Network Open.*

eTable 4. Psychological Symptoms Interaction Model Coefficients

Terms	*β*	*b*	*95% CI*	*p*
Intercept	0.00	7.76	7.64, 7.89	< .001
Age	0.03	0.03	-0.01, 0.07	.16
Male	-0.07	-0.54	-0.73, -0.35	< .001
URiM	-0.04	-0.38	-0.71, -0.06	.02
First Gen.	-0.00	-0.03	-0.32, 0.26	.85
Growth Mindset	-0.06	-0.23	-0.36, -0.10	< .001
Purpose	-0.08	-0.46	-0.68, -0.24	< .001
Belong. Uncertain.	0.45	1.34	1.23, 1.45	< .001
Age X Male	0.04	0.06	0.01, 0.11	.03
Age X URiM	-0.03	-0.07	-0.14, 0.00	.05
Age X First Gen.	0.00	0.01	-0.05, 0.07	.74
Male X URiM	0.02	0.38	-0.09, 0.85	.11
Male X First Gen.	-0.02	-0.22	-0.62, 0.18	.28
URiM X First Gen.	-0.02	-0.28	-0.78, 0.22	.28
Age X Growth Mindset	0.04	0.04	0.01, 0.08	.02
Male X Growth Mindset	0.02	0.09	-0.09, 0.27	.34
URiM X Growth Mindset	-0.01	-0.05	-0.41, 0.30	.77
First Gen. X Growth Mindset	-0.00	-0.04	-0.35, 0.28	.82
Age X Purpose	0.02	0.03	-0.04, 0.10	.38
Male X Purpose	0.00	0.02	-0.28, 0.33	.87
URiM X Purpose	-0.06	-0.91	-1.49, -0.34	.002
First Gen. X Purpose	-0.02	-0.32	-0.85, 0.21	.24
Age X Belong. Uncertain.	-0.02	-0.01	-0.05, 0.02	.44
Male X Belong. Uncertain.	0.00	-0.01	-0.17, 0.15	.91
URiM X Belong. Uncertain.	-0.01	-0.07	-0.35, 0.21	.62
First Gen. X Belong. Uncertain.	-0.00	-0.02	-0.27, 0.23	.87
Age X Male X Growth Mindset	-0.02	-0.03	-0.07, 0.02	.23
Age X URiM X Growth Mindset	0.01	0.01	-0.05, 0.07	.70
Age X First Gen. X Growth Mindset	0.00	0.00	-0.05, 0.05	.96
Male X URiM X Growth Mindset	-0.01	-0.13	-0.60, 0.34	.58
Male X First Gen. X Growth Mindset	-0.02	-0.21	-0.63, 0.21	.32
URiM X First Gen. X Growth Mindset	0.01	0.23	-0.29, 0.76	.39
Age X Male X Purpose	-0.05	-0.10	-0.19, -0.01	.03
Age X URiM X Purpose	0.03	0.08	-0.03, 0.19	.14
Age X First Generation X Purpose	-0.01	-0.03	-0.14, 0.07	.52
Male X URiM X Purpose	0.03	0.59	-0.19, 1.37	.14
Male X First Gen. X Purpose	0.02	0.34	-0.37, 1.05	.35
URiM X First Gen. X Purpose	0.05	1.37	0.47, 2.28	.003
Age X Male X Belong. Uncertainty	0.01	0.01	-0.03, 0.06	.53
Age X URiM X Belong. Uncertain.	-0.00	-0.01	-0.06, 0.05	.82
Age X First Gen. X Belong. Uncertain.	0.01	0.01	-0.04, 0.06	.70
Male X URiM X Belong. Uncertain.	0.00	0.00	-0.38, 0.38	1.00
Male X First Gen. X Belong. Uncertain.	0.00	0.04	-0.30, 0.38	.82
URiM X First Gen. X Belong. Uncertain.	0.01	0.13	-0.28, 0.54	.53

Note: R^2 = .214; *RMSE* = 4.70; URiM = underrepsented in medicine (i.e., Black/Latine/Indigenous students; Belong. Uncertain. = belonging uncertainty; *b* = unstandardized beta coefficient; β = standardized beta coefficient; *RMSE* = root mean square error

© 2024 Tibbetts Y et al. *JAMA Network Open.*

Figure 1. Ethnicity X Growth Mindset on Flourishing

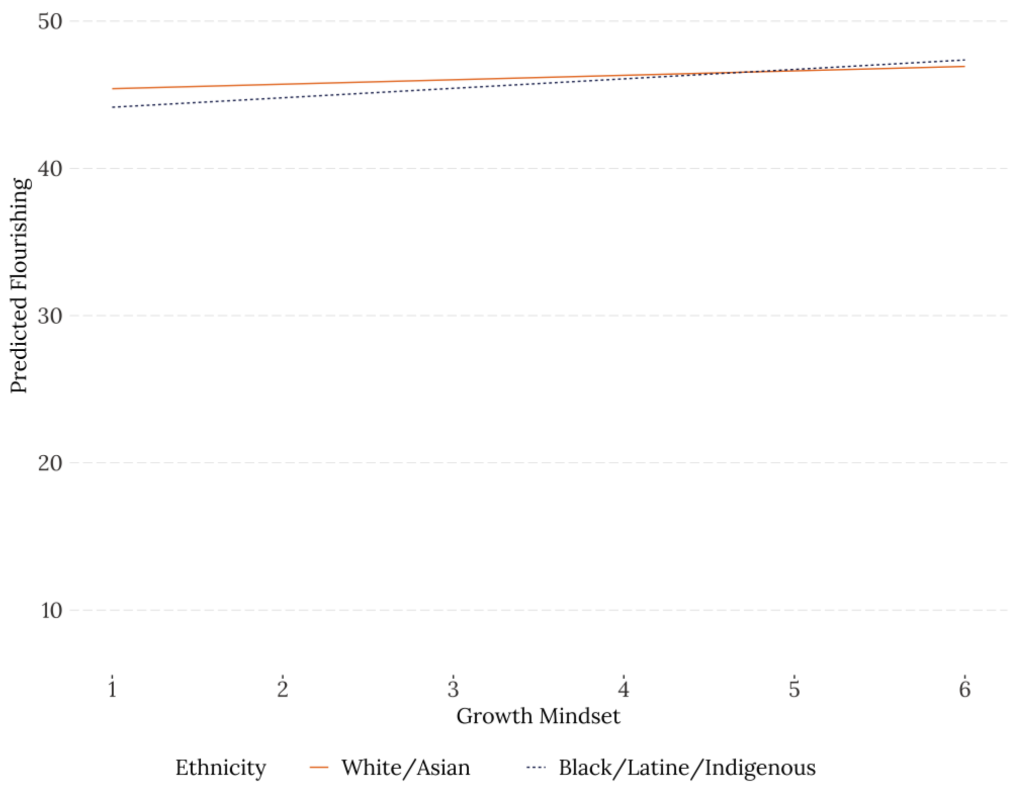

© 2024 Tibbetts Y et al. *JAMA Network Open.*

Figure 2. Gender X Purpose on Flourishing

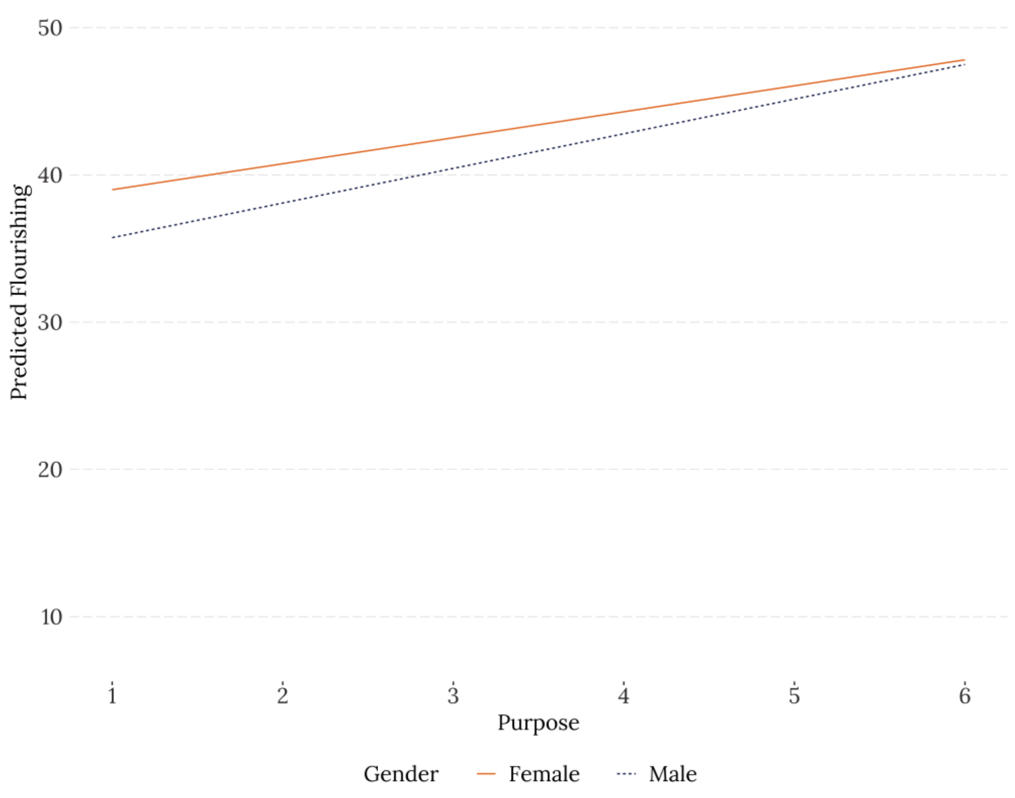

© 2024 Tibbetts Y et al. *JAMA Network Open.*

Figure 3. Ethnicity X Gender on Resilience

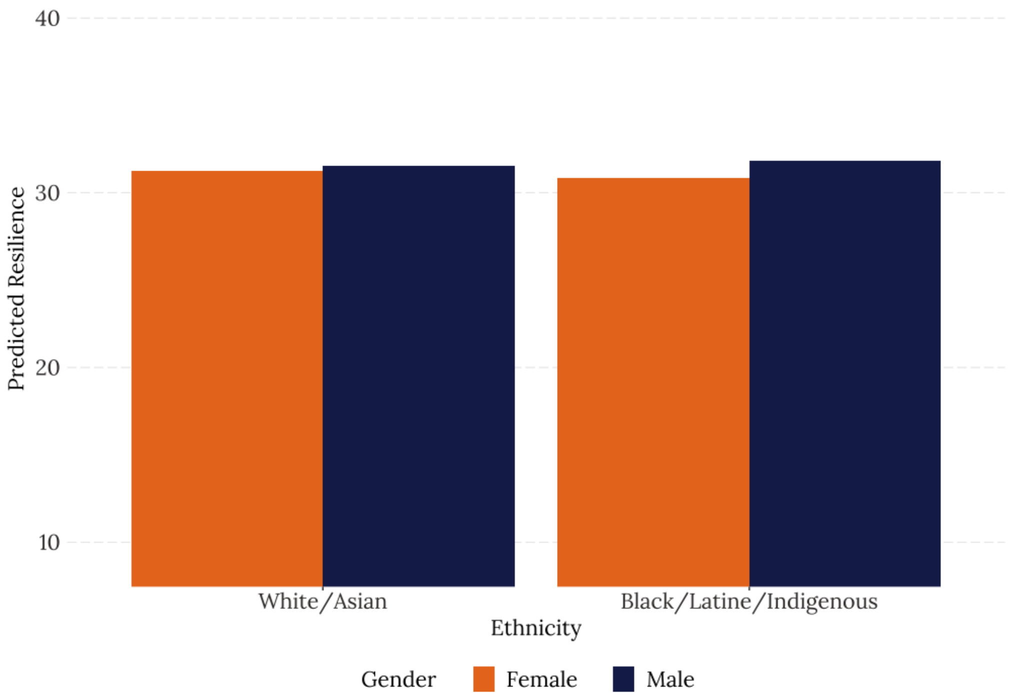

© 2024 Tibbetts Y et al. *JAMA Network Open*.

Figure 4. Gender X Growth Mindset on Resilience

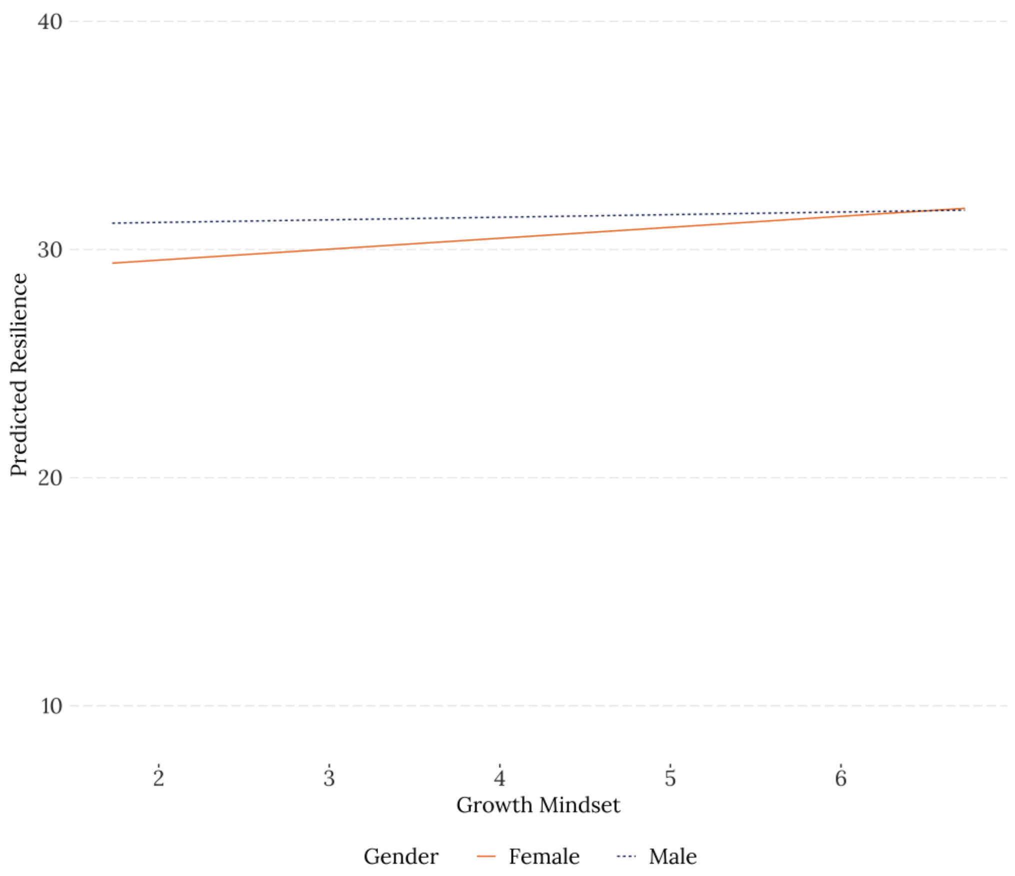

© 2024 Tibbetts Y et al. *JAMA Network Open.*

Figure 5. Gender X First Gen. Status X Purpose on Resilience

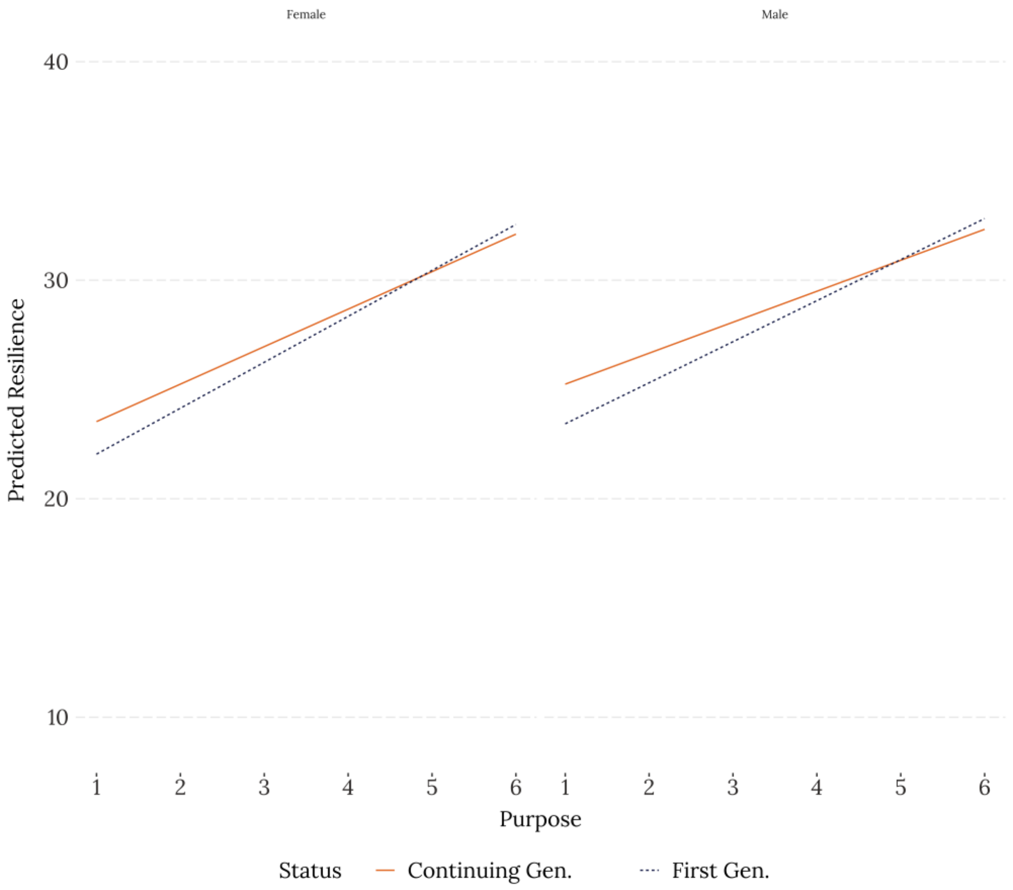

© 2024 Tibbetts Y et al. *JAMA Network Open.*

Figure 6. Age X Belonging Uncertainty on Burnout

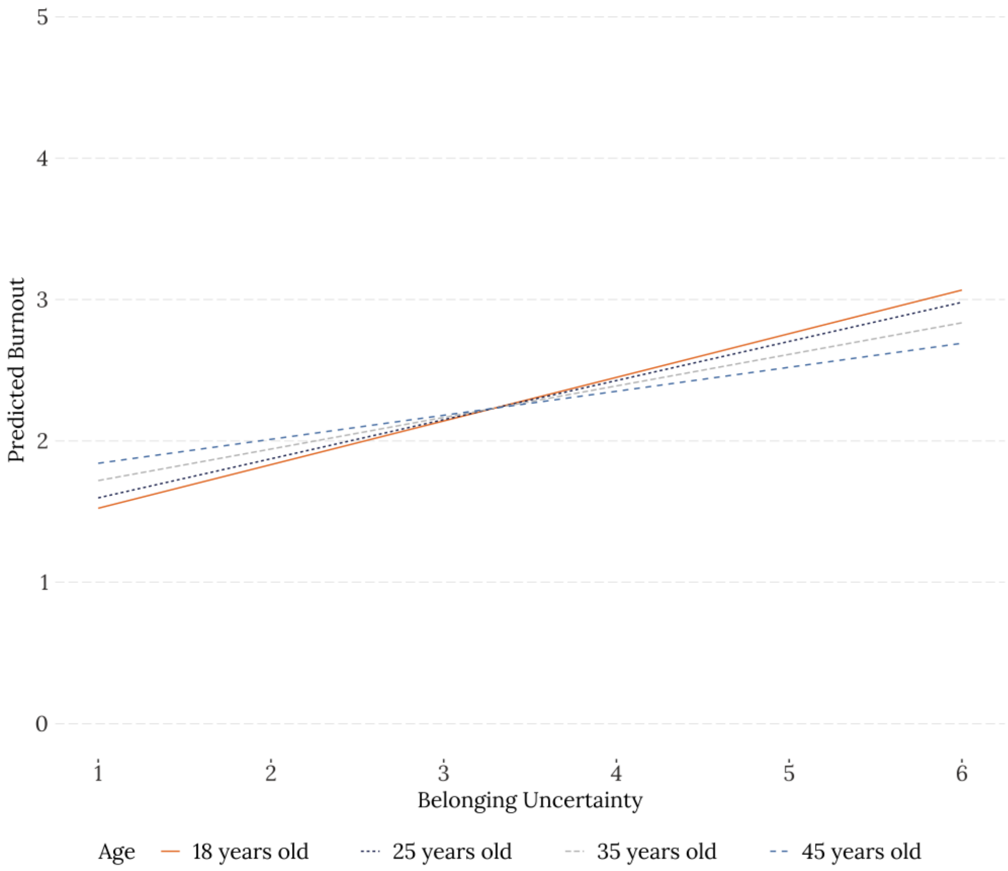

© 2024 Tibbetts Y et al. *JAMA Network Open.*

Figure 7. Age X Gender X Purpose on Burnout

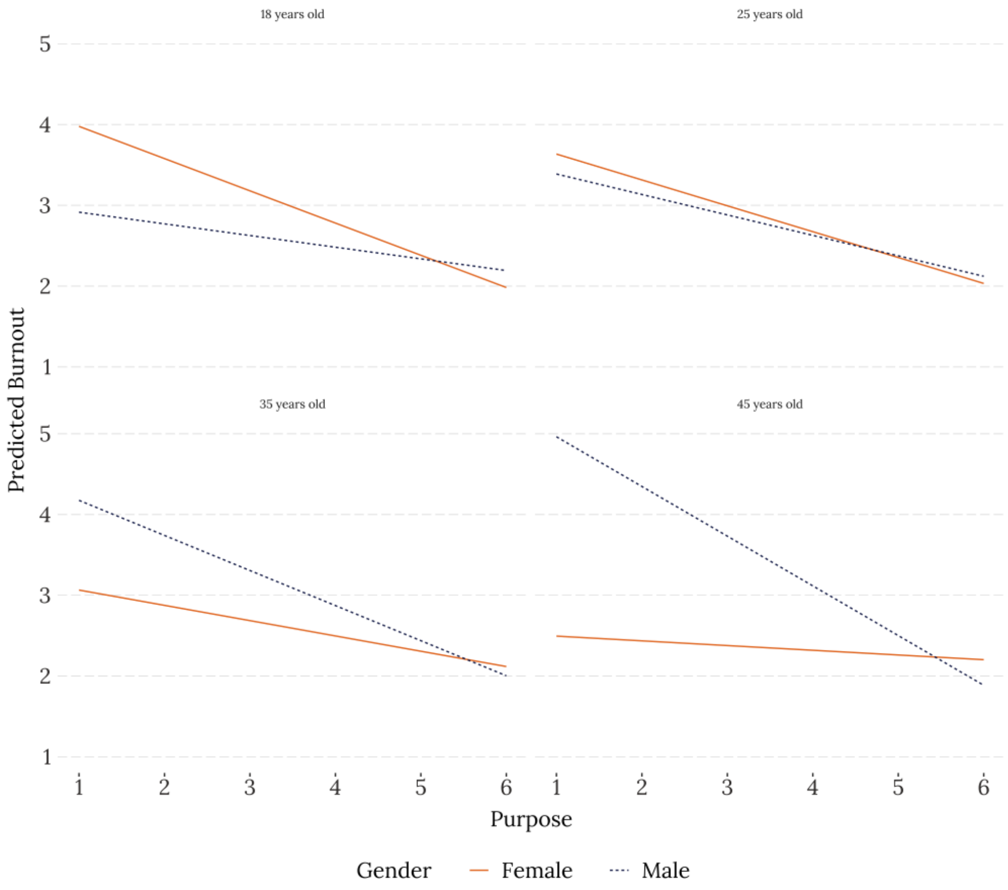

© 2024 Tibbetts Y et al. *JAMA Network Open.*

Figure 8. Gender X First Gen. Status X Purpose on Burnout

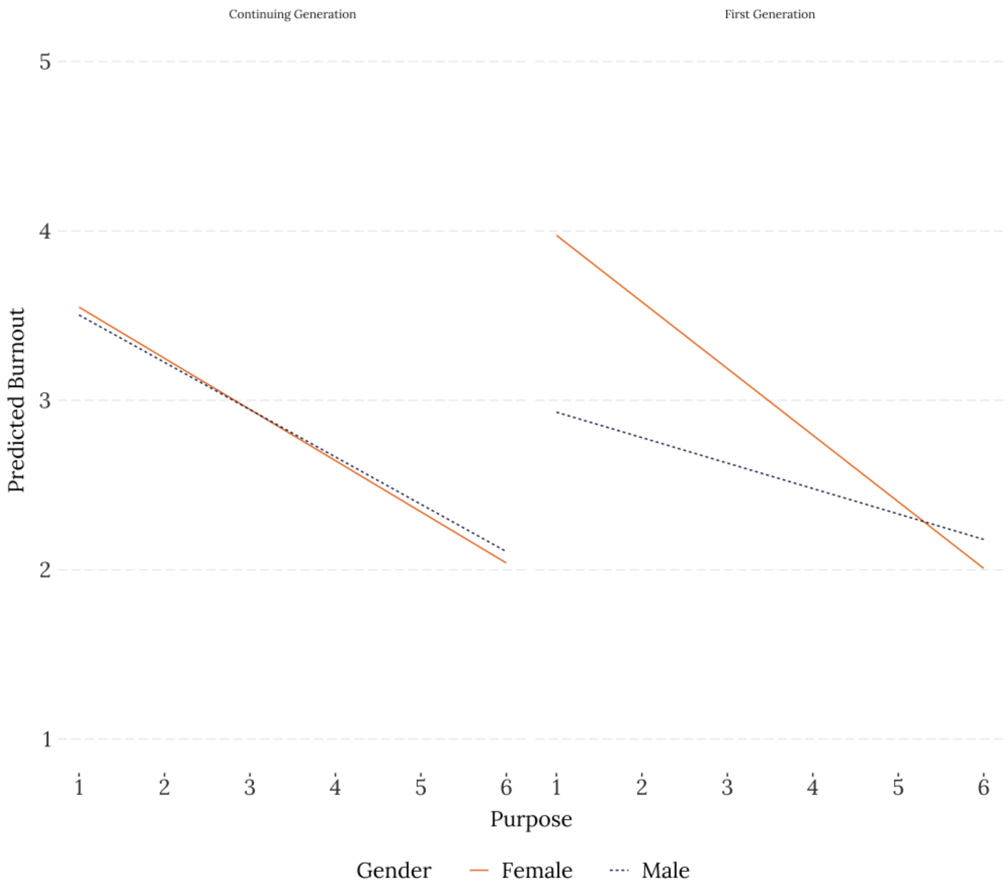

© 2024 Tibbetts Y et al. *JAMA Network Open.*

Figure 9. Age X Gender on Psychological Symptoms

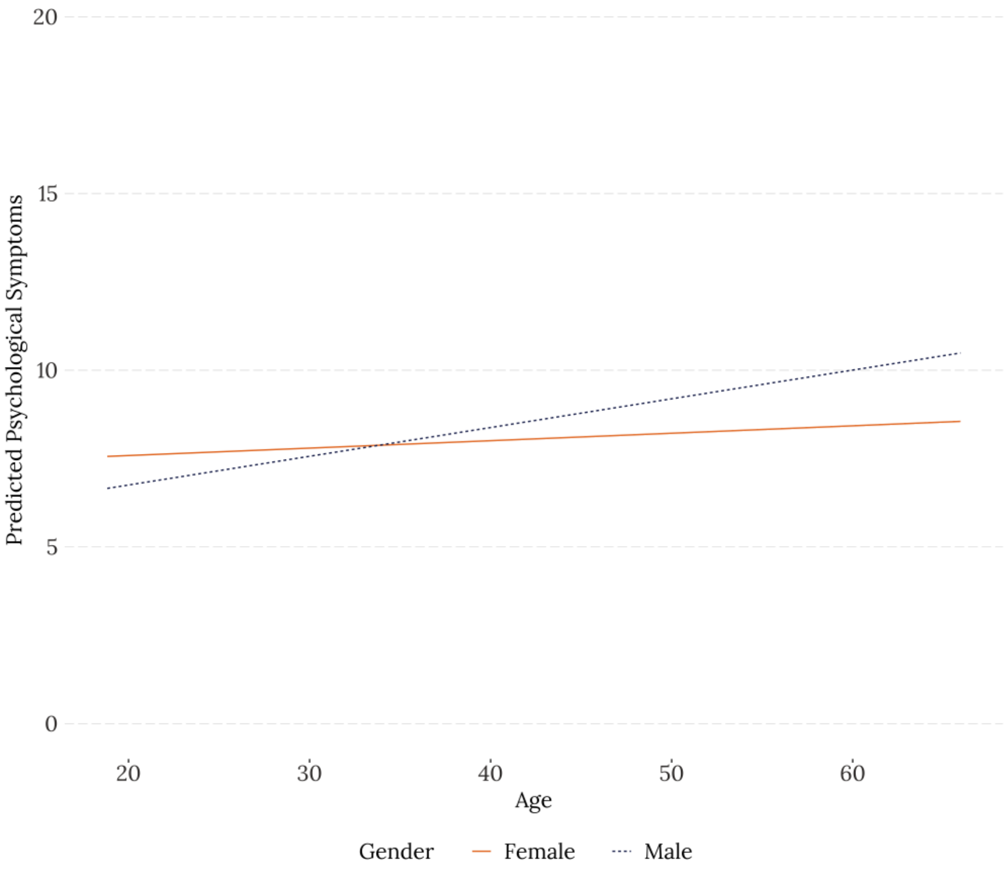

© 2024 Tibbetts Y et al. *JAMA Network Open.*

Figure 10. Age X Ethnicity on Psychological Symptoms

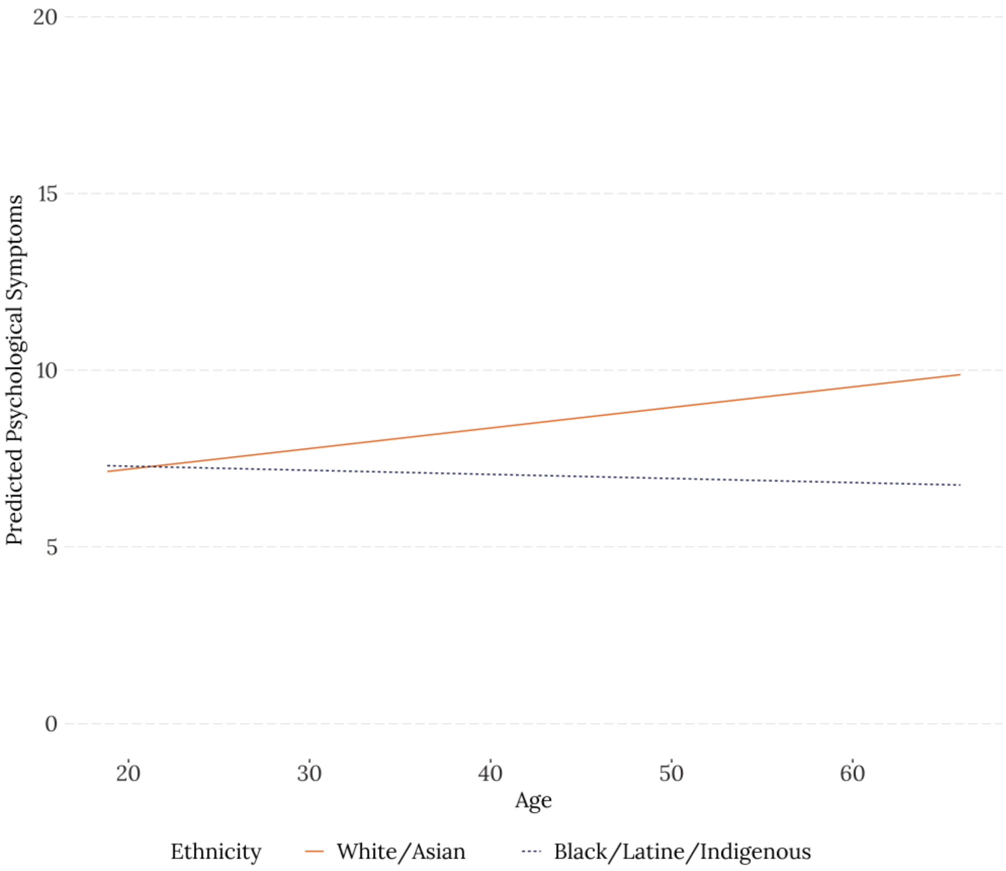

© 2024 Tibbetts Y et al. *JAMA Network Open.*

Figure 11. Age X Growth Mindset on Psychological Symptoms

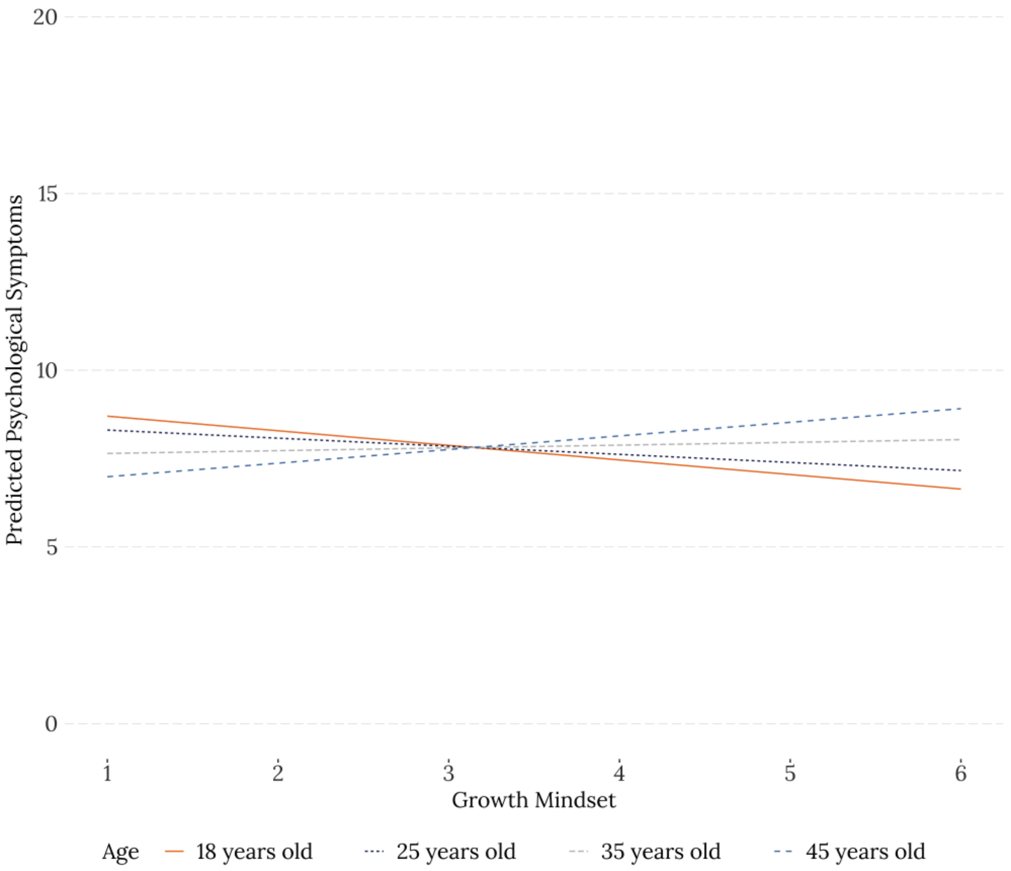

© 2024 Tibbetts Y et al. *JAMA Network Open*.

Figure 12. Ethnicity X Purpose on Psychological Symptoms

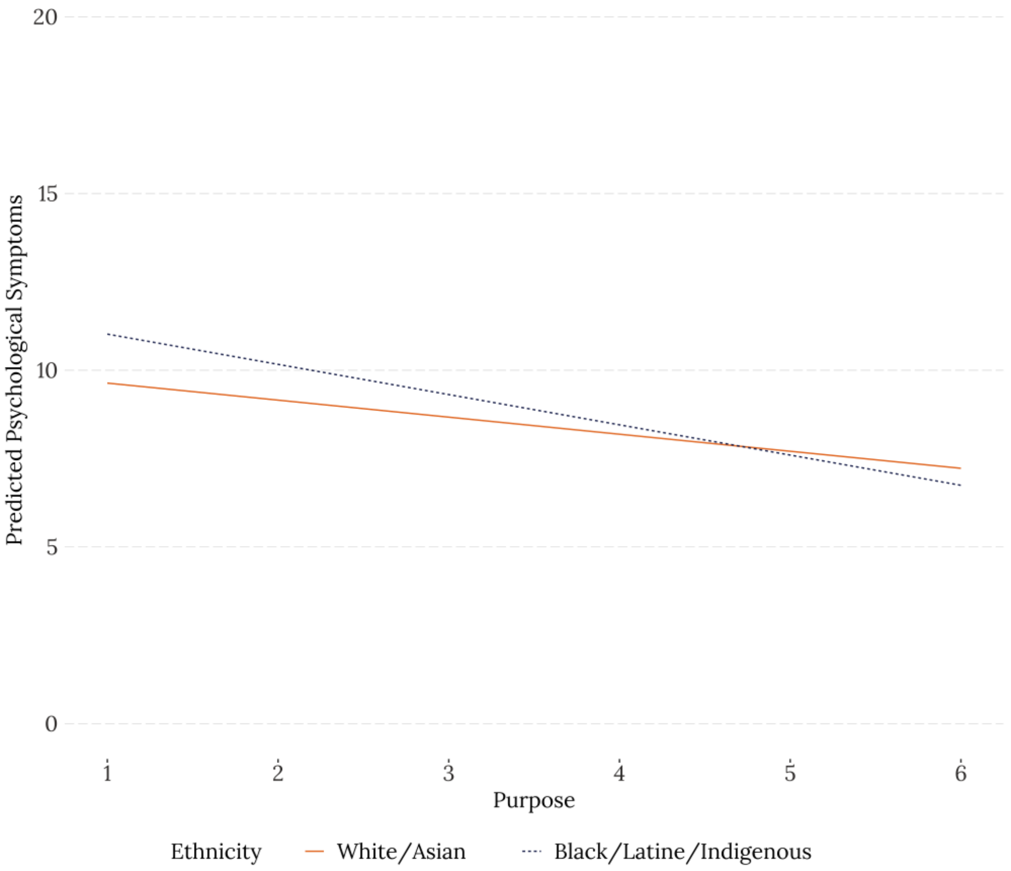

© 2024 Tibbetts Y et al. *JAMA Network Open.*

Figure 13. Age X Gender X Purpose on Psychological Symptoms

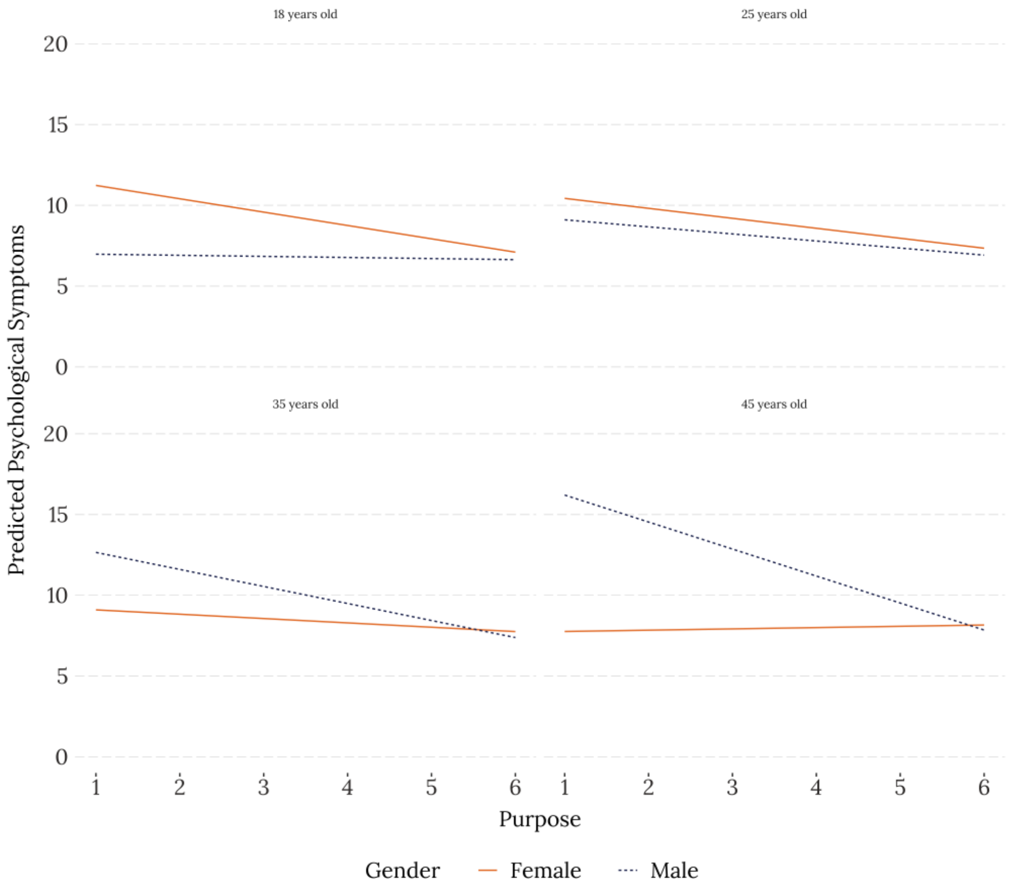

© 2024 Tibbetts Y et al. *JAMA Network Open.*

Figure 14. Ethnicity X First Gen. Status X Purpose on Psychological Symptoms

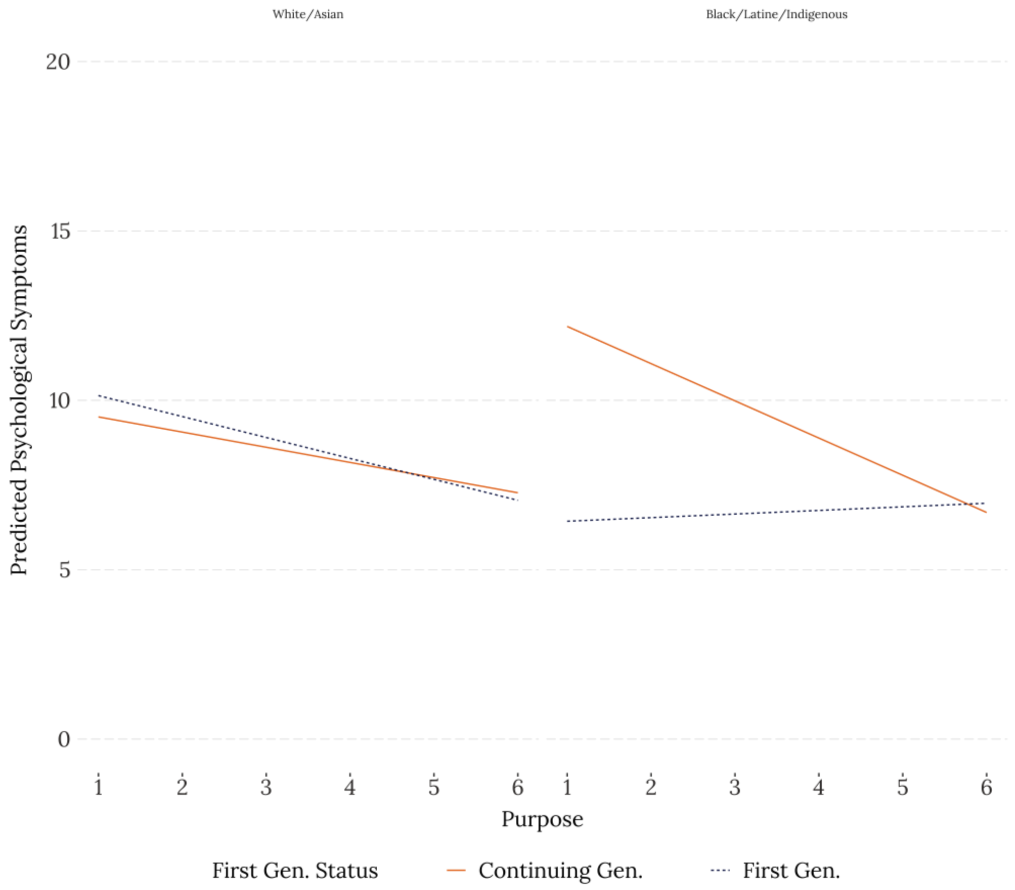

© 2024 Tibbetts Y et al. *JAMA Network Open.*
