# Supplementary material for: Learning Mindsets and Well-Being and Ill-Being Among Osteopathic Medical Students
Source: JAMA Netw Open. 2024 Jun 14;7(6):e2418090. doi: 10.1001/jamanetworkopen.2024.18090 (PMC11179131; doi:10.1001/jamanetworkopen.2024.18090)
Supplement: Supplement 2. — Data Sharing Statement [file jamanetwopen-e2418090-s002.pdf]

Data Sharing Statement

Tibbetts. Learning Mindsets and Well-Being and Ill-Being Among Osteopathic Medical Students. *JAMA Netw Open*. Published June 21, 2024.
doi:10.1001/jamanetworkopen.2024.18090

Data
Data available: Yes
Data types: Other (please specify)
Additional Information: De-identified data may be available upon request.
How to access data: Please email cyt7d@virginia.edu with data requests.
When available: With publication

Supporting Documents
Document types: None

Additional Information
Who can access the data: Researchers whose proposed use of the data has been approved.
Types of analyses: For the purposes of improving medical education.
Mechanisms of data availability: With investigator support.
